# Frameshift mutation spectra overlap between constitutional mismatch repair deficiency tumors and Lynch syndrome tumors

**DOI:** 10.1172/JCI185999

**Published:** 2024-10-22

**Authors:** Yurong Song, Ryan N. Baugher, Todd B. Young, Brandon Somerville, Yuriko Mori, Ligia A. Pinto, Kim E. Nichols, Robert H. Shoemaker

**Affiliations:** 1 Vaccine, Immunity and Cancer Directorate,; 2 Molecular Diagnostics Laboratory, and; 3 Clinical Research Directorate, Frederick National Laboratory for Cancer Research, Frederick, Maryland, USA.; 4 Division of Cancer Predisposition, St. Jude Children’s Research Hospital, Memphis, Tennessee, USA.; 5 Division of Cancer Prevention, National Cancer Institute, Bethesda, Maryland, USA.

**Keywords:** Oncology, Brain cancer, Genetic diseases

**To the Editor:** Frameshift neoantigen-based cancer prevention vaccines (e.g., Nous-209, NCT05078866) are being tested in clinical trials for patients with Lynch syndrome (LS) caused by monoallelic mismatch repair (MMR) gene pathogenic variants (PVs). A liquid biopsy biomarker panel has been developed for profiling frameshift mutations (FSMs) in tumors and plasma cell-free DNA (cfDNA) for disease surveillance of LS (1). However, it is unknown whether the same vaccine strategy and biomarker panel can be applied to patients with constitutional mismatch repair deficiency (CMMRD) with germline biallelic MMR gene PVs. CMMRD is associated with an increased risk of developing brain tumors with high frequency (51%) and has the poorest outcomes among other malignancies, starting in childhood (2). We hypothesized that those loci mutated in LS would also be frequently mutated in CMMRD malignancies and that the vaccines and biomarker panel developed for LS would be applicable to CMMRD.

To test this hypothesis, 2 patients with CMMRD with brain tumors and biallelic germline *PMS2* mutations were studied (Supplemental Table 1; supplemental material available online with this article; https://doi.org/10.1172/JCI185999DS1). Specimens were collected at the time of residual tumor resection for patient 1, at 6 years of age. For patient 2, who had glioblastoma (GBM), tumor tissue was collected at age 23, a year after blood collection.

To determine whether FSMs discovered in LS were also present in CMMRD, tumor DNA and cfDNA from 2 patients with CMMRD were sequenced using a 122-gene panel designed around loci known to be frequently mutated in LS tumors and loci corresponding to frameshift neoantigens in vaccines. A high number of FSMs were detected in tumors (Table 1 and Supplemental Table 2) (*n* = 214 [80% of genes in the panel; patient 1] vs. *n* = 52 [32% of genes in the panel; patient 2]), although patient 2 was older at the tumor collection (23 years of age) and had developed other cancers prior to GBM diagnosis. Deep allele frequency (DAF) was variable, with the highest DAF being 0.3592 for the *ASXL1* A/AG variant (Supplemental Table 3). The discrepancy in the number of FSMs detected might be due to invasive residual tumor edge tissue used for patient 1 and/or clonal evolution of tumor cells in the mass (thus, the low DAF for most FSMs [below the detection level]) for patient 2.

It is well accepted that brain tumors usually release fewer tumor fragments into the circulation owing to the blood-brain barrier. However, we observed a high number of FSMs in the blood (*n* = 43 [patient 1] vs. *n* = 47 [patient 2]) (Table 1). It is worth noting that the blood used for patient 2 was collected 1 year prior to GBM diagnosis, suggesting that FSMs may accumulate as a result of previously diagnosed tumors and/or FSMs could be detected before cancer diagnosis. Consistent with the finding from LS cfDNA (1), the number of FSMs detected in cfDNA was lower than that in tumors, especially for patient 1. The profile of FSMs was similar to what has been previously reported (3, 4) and what we have observed in LS (1). Moreover, only 1 FSM in cfDNA had a DAF of >0.05 (A*STE2* C/CT; DAF = 0.076; Supplemental Table 3), suggesting that FSMs were not enriched in cfDNA. DAF at individual loci was characteristic, and sequence context may be an important determinant.

Further analysis revealed that 94% and 42% of genes detected in cfDNA of patients 1 and 2, respectively, were shared with the paired tumor samples (Table 1). The low DAF in cfDNA may partially explain the discordance between the number of FSMs detected in blood and that detected in tumors. In addition, tumor heterogeneity, previous tumors in patient 2, and the likelihood of DNA fragments shed by all body cells in patients with CMMRD starting at a very young age may also account for the discordance.

To determine whether the number of FSMs detected in patients with CMMRD was higher than that in patients with LS, tumors from 3 young patients with LS with 2 paired cfDNA (see *Patients and specimens* in Supplemental Methods) were also sequenced using the same platform (Supplemental Table 4). The number of FSMs was much lower in LS tumors and cfDNA (*n* = 9–33 [7%–21% of genes in the panel] vs. *n* = 8–10 [7%–8% of genes in the panel], respectively), despite the presence of MMR gene loss of heterozygosity in the LS tumors. This indicates that FSMs may emerge and accumulate at a very young age from all body cells of patients with CMMRD and more DNA fragments may be shed and accumulated in the blood of patients with CMMRD than patients with LS.

It is challenging to care for patients with CMMRD because multiple primary cancers may emerge over the life course and affect a variety of different tissues. This increases the complexity of disease surveillance and management (5), which highlights the need for enhanced preventive and surveillance strategies. Our study provides evidence of the existence of FSMs in CMMRD and suggests that FSMs are not syndrome or cancer type specific. Further preclinical and clinical evaluation will be required to establish whether frameshift neoantigen-based vaccines and the biomarker panel developed for LS are effective in CMMRD.

## Supplementary Material

Supplemental data

## Figures and Tables

**Table 1:**
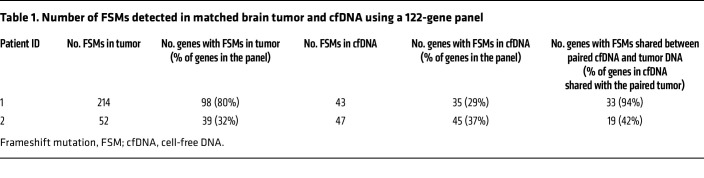
Number of FSMs detected in matched brain tumor and cfDNA using a 122-gene panel

## Version 1 10/22/2024

In-Press Preview

## Version 2 12/16/2024

Electronic publication
