# Supplementary material for: Frameshift mutation spectra overlap between constitutional mismatch repair deficiency tumors and Lynch syndrome tumors
Source: J Clin Invest. 2024 Oct 22;134(24):e185999. doi: 10.1172/JCI185999 (PMC11645132; doi:10.1172/JCI185999)
Supplement: Supplemental data [file jci-134-185999-s209.pdf]

Supplemental Material

Frameshift mutation spectra overlap between constitutional mismatch repair deficiency tumors and Lynch syndrome tumors

Yurong Song,[1*] Ryan N. Baugher,[2] Todd B. Young,[2] Brandon Somerville,[1] Yuriko Mori,[3] Ligia A. Pinto,[1] Kim E. Nichols,[4] and Robert H. Shoemaker[5]

[1]Vaccine, Immunity and Cancer Directorate, [2]Molecular Diagnostics Laboratory, [3]Clinical Research Directorate, Frederick National Laboratory for Cancer Research, Frederick, MD, USA. [4]Division of Cancer Predisposition, St. Jude Children's Research Hospital, Memphis, TN, USA. [5]Division of Cancer Prevention, National Cancer Institute, Bethesda, MD, USA.

Corresponding author
Yurong Song: songy3@mail.nih.gov

Supplemental Material Content

Supplemental Methods

Supplemental Table 1. CMMRD patient and specimen description

Supplemental Table 2. Frameshift mutations detected in tumor and cfDNA from two CMMRD patients with brain tumors

Supplemental Table 3. Sequencing depth and DAF of FSMs in brain tumors* and cfDNA**

Supplemental Table 4. Patient and specimen description and number of FSMs detected in bone marrow and cfDNA of LS patients

Supplemental Methods

Patients and specimens

Two CMMRD patients with brain tumors and biallelic germline *PMS2* mutations were included in the study (Supplemental Table 1). Deidentified tumors and paired plasma or serum samples were collected from 2 CMMRD (Supplemental Table 1) and 3 LS (Supplemental Table 4) patients at St. Jude Children's Research Hospital. The LS patients, used as a comparison group, were roughly age-matched but not tumor type-matched due to the availability of patients. LS patient LS01 had no paired plasma sample available. All patients were confirmed as CMMRD or LS based on clinical sequencing. Demographic and diagnostic information of CMMRD and LS patients were provided by the site, if available.

Targeted next generation sequencing (NGS)

Brain tumor DNA and Cell-free DNA (cfDNA) were extracted at FNLCR using Qiagen DNeasy Blood & Tissue Kit and Qiagen QIAamp Circulating Nucleic Acid Kit (Qiagen, Germantown, MD), respectively. Libraries were constructed using a starting input DNA of 100 ng and a customized 122-gene panel (Archer, now IDT, Coralville, Iowa). All samples were processed using the ArcherDx LiquidPlex Library Prep Kit and LiquidPlex ctDNA Protocol for Illumina as described previously (1). Sequencing was performed on Illumina NextSeq 2000 using NextSeq 2000 P3 Reagents (300 cycles). For background error correction, buffy coat DNA (n=10) and cfDNA (n=7) from healthy individuals were processed and sequenced the same way as they were done for tumor samples (1).

NGS data analysis

Raw FASTQ files from patients or healthy individuals were uploaded to Archer® Analysis Virtual Machine with a NGS data analysis pipeline developed by Archer (Archer® Analysis Virtual Machine 7.2, Archer, now IDT, Coralville, Iowa) for data analysis as described previously (1). Sequencing data from healthy individuals were analyzed and used as normal dataset for background error correction. ND DAF (Normal Dataset Detectable Allele Fraction) Outlier P-value (the probability that this mutation was due to background noise given the provided Normal Dataset and unique molecules with a deep amplicon depth taken into account) was computed using Archer® Analysis and positive mutation calling was made when $P < 0.05$.

Sex as a biological variable

Our study examined male and female patients, and similar findings are reported for both sexes. Due to the disease rarity, all patients with samples available were included in the study.

Study Approval

Specimens were collected after patients provided written informed consent in accordance with institutional protocols and study was approved by the institutional review board at St. Jude Children's Research Hospital (Federal Wide Assurance: FWA00004775). The use of deidentified biospecimens was reviewed and determined exempt by Frederick National Laboratory of Cancer Research (FNLCR) and the study was conducted in accordance with the U.S. Common Rule.

Data availability

The raw data generated in this study are available for download via the National Center for Biotechnology Information Sequence Read Archive at https://www.ncbi.nlm.nih.gov/sra/PRJNA1140057 and can be accessed with BioProject accession No. PRJNA1140057.

Acknowledgements

We thank the patients and their families for participating in this research. We acknowledge the St. Jude Biorepository at St. Jude Children's Research Hospital, Inc. for providing the samples. We thank Dr. Rachana Agarwal at the Clinical Research Directorate and Drs. Daniel R. Soppet and Stephen Hewitt and Kristen M. Pike at the CLIA Molecular Diagnostics Laboratory for project support, and Cancer Immunoprevention lab members and scientists at the Chemopreventive Agent Development Research Group at National Cancer Institute, Division of Cancer Prevention, for data discussion. This project has been funded in part with Federal funds from the National Cancer Institute, National Institutes of Health, Department of Health and Human Services, under Contract No. 75N91019D00024. The content of this publication does not necessarily reflect the views or policies of the Department of Health and Human Services, nor does mention of trade names, commercial products, or organizations imply endorsement by the U.S. Government.

Author contributions

Y.S. and R.H.S. conceptualized the project and drafted the manuscript. Y.S. analyzed and interpreted sequencing data. R.N.B. and T.B.Y. performed library construction and NGS sequencing. B.S. processed the biospecimens. Y.M. procured samples. L.A.P. reviewed the manuscript. K.E.N. collected the clinical data, provided tumor and plasma samples, and edited the manuscript. All authors reviewed and approved the manuscript.

References

1. Song Y, Loomans-Kropp H, Baugher RN, Somerville B, Baxter SS, Kerr TD, et al. Frameshift mutations in peripheral blood as a biomarker for surveillance of Lynch syndrome. *J Natl Cancer Inst.* 2024;116(6):957-65.

Supplemental Tables

Supplemental Table 1. CMMRD patient and specimen description

Characteristics and specimen	Patient 1	Patient 2
Syndrome	CMMRD	CMMRD
Affected gene	*PMS2*	*PMS2*
Germline mutation	c.765C>A; c.736_741delinsTGTGTGTGAAG	c.1239_1240insA; c.1927C>T
Gender	Female	Male
Race	White	Black
Diagnosis	Residual high-grade glioma	GBM, giant cell variant
Brain tumor staging	WHO grade III or IV	WHO grade IV
Age at Diagnosis	6	14
Age at sample Collection	6	23
Collection Date in reference to diagnosis date	27 days	9 years
Therapy prior to sample collection	None	Resection, RT, Chemotherapy
Clinical History	Main tumor mass was resected 27 days prior to the residual tumor sample collection. The sample was collected during 2nd resection performed at St. Jude due to the presence of the residual tumor detected upon a follow-up visit after the initial surgery*	Dx at age 14 with anaplastic astrocytoma s/p resection/RT/chemo. Dx with adenocarcinoma of duodenum at age 15 treated with resection & chemo. Dx with glioblastoma (GBM) at age 23 s/p resection & RT. Dx age at 24 with basal cell carcinoma s/p excision. Dx at age 25 with high grade astrocytoma s/p excision
Blood Specimen (amount available)	Plasma (1 mL)	Serum (from one year earlier at age 22; 0.5 mL)**
Tissue Specimen available	Residual brain tumor tissue	GBM tumor tissue at age 23

*No further details regarding grade are available for this tumor sample. A high-grade glioma is by default WHO grade III (anaplastic astrocytoma) or IV (glioblastoma multiforme; GBM).
**Previous cancers were in remission at the time of blood collection at age 22.
RT: Radiotherapy; Dx: diagnosis; s/p: status post.

Supplemental Table 2. Frameshift mutations detected in tumor and cfDNA from two CMMRD patients with brain tumors

FSM	Patient #1_ Tumor	Patient #2_ Tumor	Patient #1_ Plasma cfDNA	Patient #2_ Serum cfDNA	*Grand Total*
ABCC5(+1)_C / CA_10	1				*1*
ADD3(+1)_C / CA_9	1				*1*
ADD3(-1)_CA / C_8	1	1		1	*3*
ADNP(+1)_C / CT_9	1			1	*2*
ADNP(-1)_CT / C_8	1	1			*2*
AIM2(+1)_G / GT_11	1	1			*2*
AIM2(+2)_G / GTT_12	1				*1*
AIM2(-1)_GT / G_10	1	1			*2*
AKAP7(-1)_GA / G_8	1				*1*
ARV1(+1)_C / CA_10	1	1			*2*
ARV1(-1)_CA / C_9	1				*1*
ARV1(-2)_CAA / C_9	1				*1*
ASTE1(+1)_C / CT_12	1		1		*2*
ASTE1(+2)_C / CTT_13	1				*1*
ASTE1(-1)_CT / C_11	1			1	*2*
ASTE1(-2)_CTT / C_11	1				*1*
ASXL1(+1)_A / AG_9	1	1			*2*
ASXL1(+2)_A / AGG_10	1				*1*
ATAD2(-1)_CT / C_9	1				*1*
BAX(+1)_T / TG_9	1		1	1	*3*
BAX(-1)_TG / T_8	1		1		*2*
BMPR2(-1)_GA / G_7	1			1	*2*
BRD3(+1)_C / CG_9	1				*1*
BRD3(+2)_C / CGG_10	1				*1*
CCDC168(+1)_C / CA_10	1				*1*
CCDC168(+2)_C / CAA_11	1				*1*
CCDC168(-1)_CA / C_9	1		1		*2*
CCDC28A(+1)_C / CA_9	1				*1*
CCDC43(-1)_CT / C_9	1				*1*
CCDC43(-1)_TC / T_9	1				*1*
CCDC73(+1)_A / AT_10	1	1			*2*
CCDC73(-1)_AT / A_9	1		1	1	*3*
CD3G(+1)_T / TA_10	1				*1*
CD3G(+2)_T / TAA_11	1				*1*
CD3G(-1)_TA / T_9	1	1			*2*
CD3G(-2)_TAA / T_9	1				*1*
CDC7(+1)_T / TA_10	1				*1*
CDC7(-1)_TA / T_9	1			1	*2*
CEBPZ(+1)_C / CT_10	1	1			*2*
CEBPZ(-1)_CT / C_9	1				*1*
CEP290(+1)_A / AT_11	1				*1*
CEP290(+2)_A / ATT_12	1				*1*
CEP290(-1)_AT / A_10	1				*1*
CEP290(-2)_ATT / A_10	1				*1*
CKAP2(+1)_G / GA_9	1				*1*
CKAP2(-1)_GA / G_8	1	1			*2*
COBLL1(+1)_C / CA_10	1				*1*
DNA2(+1)_G / GA_10	1				*1*
DNA2(-1)_AG / A_9	1				*1*
DNA2(-1)_GA / G_9	1	1		1	*3*
DNA2(-2)_GAA / G_9	1				*1*

Supplemental Table 2. (Continued)

FSM	Patient #1_ Tumor	Patient #2_ Tumor	Patient #1_ Plasma cfDNA	Patient #2_ Serum cfDNA	*Grand Total*
DYNC1I2(-1)_GA / G_9	1		1		***2***
DYNC1I2(-2)_GAA / G_9	1				***1***
EIF2B3(-1)_CT / C_9	1	1	1	1	***4***
EIF2B3(-2)_CTT / C_9	1				***1***
EIF3J(-1)_GA / G_8	1			1	***2***
FAM214A(-1)_AT / A_8	1		1		***2***
FBXL3(+1)_T / TA_10	1				***1***
FBXL3(+2)_T / TAA_11	1				***1***
FBXL3(-1)_TA / T_9	1		1		***2***
FHOD3(-1)_AC / A_6	1	1	1	1	***4***
GBP3(+1)_G / GT_11	1				***1***
GBP3(+2)_G / GTT_12	1				***1***
GBP3(-1)_GT / G_10	1				***1***
GRB14(-1)_GT / G_9	1	1	1		***3***
ICA1(-1)_GT / G_9	1		1		***2***
JPH4(+1)_G / GC_10	1				***1***
JPH4(+2)_G / GCC_11	1				***1***
JPH4(-2)_GCC / G_9	1				***1***
KCNMA1(+1)_C / CT_10	1				***1***
KCNMA1(-1)_CT / C_9	1	1		1	***3***
KCNMA1(-2)_CTT / C_9	1				***1***
KCTD16(+1)_C / CA_10	1				***1***
KCTD16(+2)_C / CAA_11	1				***1***
KCTD16(-1)_CA / C_9	1				***1***
KIAA1024(+1)_T / TA_9	1				***1***
KIAA1024(-1)_TA / T_8	1	1		1	***3***
KMT2C(+1)_C / CT_10	1		1		***2***
KMT2C(-1)_CT / C_9	1				***1***
KNOP1(+1)_A / AT_11	1				***1***
KNOP1(-1)_AT / A_10	1				***1***
LARP7(+1)_T / TA_9	1		1		***2***
LARP7(+2)_T / TAA_10	1				***1***
LARP7(-1)_TA / T_8	1				***1***
LMAN1(+1)_C / CT_10	1				***1***
LMAN1(+2)_C / CTT_11	1				***1***
LMAN1(-1)_CT / C_9	1			1	***2***
LTN1(+1)_A / AT_12	1				***1***
LTN1(+2)_A / ATT_13	1				***1***
LTN1(-1)_AT / A_11	1		1	1	***3***
LTN1(-2)_ATT / A_11	1				***1***
MDN1(+1)_C / CT_9	1				***1***
MDN1(-1)_CT / C_8	1				***1***
MLH3(-1)_CT / C_9	1		1		***2***
MSH3(-1)_AG / A_8	1				***1***
MSH3(-1)_CA / C_8	1				***1***
NBEAL1(+1)_C / CA_10	1				***1***
NBEAL1(+2)_C / CAA_11	1				***1***
NBEAL1(-1)_CA / C_9	1			1	***2***
NDUFC2(+1)_C / CA_10	1				***1***
NDUFC2(-1)_CA / C_9	1	1	1	1	***4***
NDUFC2(-2)_CAA / C_9	1	1			***2***
NOL4L(+1)_A / AG_9	1				***1***
NOL4L(+2)_A / AGG_10	1				***1***
NOL4L(-1)_AG / A_8	1				***1***
PARP14(+1)_G / GA_11	1				***1***
PARP14(+2)_G / GAA_12	1				***1***
PARP14(-1)_GA / G_10	1				***1***
PHACTR4(+1)_G / GA_11	1				***1***
PHACTR4(-1)_GA / G_10	1				***1***

Supplemental Table 2. (Continued)

FSM	Patient #1_ Tumor	Patient #2_ Tumor	Patient #1_ Plasma cfDNA	Patient #2_ Serum cfDNA	*Grand Total*
PLEKHA6(+1)_A / AC_7	1				*1*
PRDM2(-1)_CA / C_9	1	1		1	*3*
PRR11(+1)_C / CA_10	1			1	*2*
PRR11(-1)_CA / C_9	1				*1*
PRRG1(+1)_A / AC_9	1		1		*2*
PRRT2(-1)_GC / G_9	1	1			*2*
RAD50(+1)_T / TA_10	1	1			*2*
RAD50(+2)_T / TAA_11	1	1			*2*
RAD50(-1)_TA / T_9	1	1	1	1	*4*
RBM27(+1)_T / TA_10	1				*1*
RBM27(+2)_T / TAA_11	1				*1*
RBM27(-1)_TA / T_9	1	1		1	*3*
RFC3(+1)_T / TA_11	1	1			*2*
RFC3(+2)_T / TAA_12	1	1			*2*
RFC3(-1)_TA / T_10	1				*1*
RGS12(-1)_GA / G_9	1				*1*
RGS22(+1)_A / AT_10	1		1		*2*
RGS22(-1)_AT / A_9	1			1	*2*
RNF43(+1)_A / AC_8	1				*1*
RPL22(+1)_G / GC_7	1				*1*
SEC63(+1)_C / CT_10	1			1	*2*
SEC63(+1)_G / GT_11	1				*1*
SEC63(+2)_C / CTT_11	1				*1*
SEC63(-1)_CT / C_9	1				*1*
SEC63(-1)_GT / G_10	1		1		*2*
SETD1B(+1)_A / AC_9	1		1	1	*3*
SETD1B(-1)_AC / A_8	1		1	1	*3*
SETD1B(-1)_CA / C_10	1				*1*
SLAMF1(+1)_C / CT_10	1	1	1		*3*
SLAMF1(-1)_CT / C_9	1	1			*2*
SLC22A9(+1)_C / CA_12	1	1			*2*
SLC22A9(+2)_C / CAA_13	1	1			*2*
SLC22A9(-1)_CA / C_11	1		1		*2*
SLC35F5(+1)_C / CA_11	1	1			*2*
SLC35F5(+2)_C / CAA_12	1	1			*2*
SLC35F5(-1)_CA / C_10	1				*1*
SLC35G2(-1)_GA / G_9	1			1	*2*
SMAP1(+1)_G / GA_11	1	1			*2*
SMAP1(+2)_G / GAA_12	1	1			*2*
SMAP1(-1)_GA / G_10	1	1			*2*
SPAG9(-1)_CT / C_9	1			1	*2*
SPINK5(+1)_G / GA_11	1				*1*
SPINK5(+2)_G / GAA_12	1				*1*
SPINK5(-1)_GA / G_10	1	1		1	*3*
SREK1IP1(+1)_C / CT_11	1		1		*2*
SREK1IP1(+2)_C / CTT_12	1				*1*
SREK1IP1(-1)_CT / C_10	1				*1*
SRPRA(+1)_C / CT_9	1				*1*
SRPRA(+1)_T / TC_7	1				*1*
SRPRA(-1)_CT / C_8	1	1	1	1	*4*
STAMBPL1(-1)_TA / T_8	1				*1*
SVIL(+1)_T / TC_8	1				*1*
SVIL(-1)_TC / T_7	1			1	*2*
SYCP2(+2)_G / GTT_11	1	1			*2*
SYCP2(-1)_GT / G_9	1				*1*
SYNJ2(+1)_A / AC_9	1		1		*2*
SYNJ2(+2)_A / ACC_10	1				*1*
SYNJ2(-2)_ACC / A_8	1	1			*2*

Supplemental Table 2. (Continued)

FSM	Patient #1_ Tumor	Patient #2_ Tumor	Patient #1_ Plasma cfDNA	Patient #2_ Serum cfDNA	*Grand Total*
TAF1B(+1)_T / TA_12	1				***1***
TAF1B(+2)_T / TAA_13	1				***1***
TAF1B(-1)_TA / T_11	1				***1***
TAF1B(-2)_TAA / T_11	1				***1***
TBC1D23(+2)_C / CAA_11	1				***1***
TBC1D23(-1)_CA / C_9	1			1	***2***
TBC1D23(-2)_CAA / C_9	1				***1***
TCF7L2(+1)_G / GA_10	1		1		***2***
TCF7L2(-1)_GA / G_9	1				***1***
TEAD2(+1)_T / TG_9	1				***1***
TGFBR2(+1)_G / GA_11	1				***1***
TGFBR2(+2)_G / GAA_12	1				***1***
TGFBR2(-1)_GA / G_10	1			1	***2***
TGFBR2(-2)_GAA / G_10	1				***1***
THAP5(+1)_A / AT_10	1				***1***
THAP5(-1)_AT / A_9	1				***1***
TMEM60(-1)_CT / C_9	1		1		***2***
TMEM60(-2)_CTT / C_9	1				***1***
TNKS2(+1)_C / CA_10	1	1			***2***
TNKS2(+2)_C / CAA_11	1		1		***2***
TNKS2(-1)_CA / C_9	1				***1***
TRAPPC8(+1)_C / CA_9	1				***1***
TRAPPC8(-1)_CA / C_8	1			1	***2***
TTK(+2)_G / GAA_11	1				***1***
TTK(-1)_GA / G_9	1	1		1	***3***
TTLL10(+1)_C / CG_9	1				***1***
TTLL10(-1)_CG / C_8	1				***1***
UBR5(-1)_CT / C_8	1				***1***
UPF3A(+1)_C / CA_10	1				***1***
UPF3A(-1)_CA / C_9	1			1	***2***
USP35(+1)_C / CA_8	1				***1***
UVRAG(+1)_G / GA_11	1				***1***
UVRAG(-1)_GA / G_10	1				***1***
VCP(+1)_A / AT_10	1				***1***
VCP(+2)_A / ATT_11	1				***1***
VCP(-1)_AT / A_9	1				***1***
WDTC1(+1)_T / TG_9	1				***1***
XYLT2(+1)_A / AC_8	1	1			***2***
XYLT2(-1)_AC / A_7	1				***1***
ZBTB20(+1)_A / AG_8	1				***1***
ZBTB20(-1)_AG / A_7	1				***1***
ZDBF2(+1)_T / TA_10	1				***1***
ZDBF2(+2)_T / TAA_11	1		1		***2***
ZNF106(-1)_GA / G_8	1				***1***
ZNF365(+1)_T / TA_9	1				***1***
ZNF365(-1)_TA / T_8	1			1	***2***
CCDC73(+2)_A / ATT_11		1			***1***
DOCK3(+1)_A / AC_8		1	1		***2***
ICA1(+1)_G / GT_10		1			***1***
KDM5A(-1)_CT / C_8		1			***1***
MFSD14A(+1)_T / TA_8		1			***1***
PRRG1(-1)_AC / A_8		1			***1***
SYNJ2(-1)_AC / A_8		1	1	1	***3***

Supplemental Table 2. (Continued)

FSM	Patient #1_ Tumor	Patient #2_ Tumor	Patient #1_ Plasma cfDNA	Patient #2_ Serum cfDNA	*Grand Total*
SYNJ2(-1)_CT / C_10		1	1		***2***
WDR55(-1)_CA / C_8		1		1	***2***
ZDBF2(-1)_TA / T_9		1	1		***2***
DOCK3(-1)_AC / A_7			1	1	***2***
GRIK2(-1)_CA / C_8			1		***1***
PRRT2(+1)_G / GC_10			1		***1***
SEC63(+2)_G / GTT_12			1		***1***
SLAMF1(-1)_GT / G_4			1		***1***
TTK(-1)_GA / G_7			1		***1***
USP35(-1)_AC / A_8			1	1	***2***
ABCC5(-1)_CA / C_9				1	***1***
CCDC28A(-1)_CA / C_8				1	***1***
COBLL1(-1)_CA / C_9				1	***1***
EBPL(+2)_T / TAA_11				1	***1***
JPH4(-1)_GC / G_9				1	***1***
MFSD14A(-1)_CT / C_8				1	***1***
USP35(+1)_A / AC_9				1	***1***
Grand Total	***214***	***52***	***43***	***47***	***356***

Supplemental Table 3. Sequencing depth and DAF of FSMs in brain tumors* and cfDNA**

Symbol	HGVSp	Depth	DAF	Genomic Location	HRUN	Ref/Alt Allele	Patient ID
*ASXL1**	NP_056153.2:p.Gly646TrpfsTer12	12770	0.3592	chr20:31022441	9	A / AG	1
*JPH4**	NP_115828.2:p.Ala502GlyfsTer12	13851	0.2973	chr14:24040435	10	G / GC	1
*CCDC73**	NP_001008392.2:p.Asn638LysfsTer9	2737	0.2942	chr11:32635950	10	A / AT	2
*SLC22A9**	NP_543142.2:p.Pro336ThrfsTer5	2738	0.1821	chr11:63149670	12	C / CA	2
*KNOP1**	NP_001013009.2:p.Ile218AsnfsTer23	3099	0.1389	chr16:19725705	11	A / AT	1
*SMAP1**	NP_068759.2:p.Glu146GlyfsTer19	1536	0.128	chr6:71508369	11	G / GA	2
*LTN1**	NP_056380.3:p.Asn536LysfsTer3	17276	0.1196	chr21:30339205	12	A / AT	1
*TAF1B**	NP_005671.3:p.Asn66LysfsTer4	13759	0.111	chr2:9989570	12	T / TA	1
*RFC3**	NP_002906.1:p.Ile82AsnfsTer2	2753	0.1109	chr13:34398062	11	T / TA	2
*SLC22A9**	NP_543142.2:p.Pro336ThrfsTer5	14007	0.1011	chr11:63149670	12	C / CA	1
*SLC35F5**	NP_079457.2:p.Cys248LeufsTer24	3013	0.0989	chr2:114500276	11	C / CA	2
*RFC3**	NP_002906.1:p.Ile82AsnfsTer2	19837	0.0828	chr13:34398062	11	T / TA	1
*GBP3**	NP_060754.2:p.Thr585AsnfsTer8	19148	0.0815	chr1:89473441	11	G / GT	1
*ASTE1**	NP_054784.2:p.Arg632LysfsTer11	16848	0.0771	chr3:130733046	12	C / CT	1
*SLC35F5**	NP_079457.2:p.Cys248LeufsTer24	16282	0.0771	chr2:114500276	11	C / CA	1
*RAD50**	NP_005723.2:p.Glu723GlyfsTer5	3163	0.0701	chr5:131931451	10	T / TA	2
*ASTE1**	NP_054784.2:p.Arg632GlyfsTer33	18469	0.0697	chr3:130733046	11	CT / C	1
*PARP14**	NP_060024.2:p.Asn1322LysfsTer21	17728	0.0686	chr3:122433231	11	G / GA	1
*CEP290**	NP_079390.3:p.Ile556AsnfsTer20	19061	0.0684	chr12:88512304	11	A / AT	1
*AIM2**	NP_004824.1:p.Thr343AsnfsTer5	21641	0.0673	chr1:159032486	11	G / GT	1
*PRRT2**	NP_660282.2:p.Arg217GlufsTer12	1367	0.0667	chr16:29825015	9	GC / G	2
*KNOP1**	NP_001013009.2:p.Ile218SerfsTer41	3607	0.0625	chr16:19725705	10	AT / A	1
*SPINK5**	NP_006837.2:p.Lys824GlufsTer4	19288	0.0621	chr5:147499874	11	G / GA	1
*SMAP1**	NP_068759.2:p.Glu146GlyfsTer19	11512	0.0616	chr6:71508369	11	G / GA	1
*ICA1**	NP_004959.2:p.Asn204LysfsTer3	3263	0.0608	chr7:8198250	10	G / GT	2
*UVRAG**	NP_003360.2:p.Ser237LysfsTer2	9917	0.0602	chr11:75694430	11	G / GA	1
*TGFBR2**	NP_003233.4:p.Pro129AlafsTer3	17141	0.0594	chr3:30691871	11	G / GA	1
*SLC22A9**	NP_543142.2:p.Lys335AsnfsTer67	15697	0.0585	chr11:63149670	11	CA / C	1
*AIM2**	NP_004824.1:p.Thr343AsnfsTer5	3722	0.0568	chr1:159032486	11	G / GT	2
*THAP5**	NP_872335.2:p.Ser58IlefsTer9	5585	0.0512	chr7:108205525	10	A / AT	1
*ASTE1***	NP_054784.2:p.Arg632LysfsTer11	2903	0.076	chr3:130733046	12	C / CT	1
*ASTE1***	NP_054784.2:p.Arg632GlyfsTer33	1090	0.0488	chr3:130733046	11	CT / C	2
*SLC22A9***	NP_543142.2:p.Lys335AsnfsTer67	3067	0.0465	chr11:63149670	11	CA / C	1
*SPINK5***	NP_006837.2:p.Lys823ArgfsTer101	998	0.0417	chr5:147499874	10	GA / G	2
*SREK1IP1***	NP_776190.1:p.Arg91LysfsTer11	2231	0.0392	chr5:64023940	11	C / CT	1
*KMT2C***	NP_733751.2:p.Glu2798GlyfsTer11	1192	0.0378	chr7:151874147	10	C / CT	1
*RAD50***	NP_005723.2:p.Lys722ArgfsTer14	1262	0.032	chr5:131931451	9	TA / T	2
*SEC63***	NP_009145.1:p.Lys535AsnfsTer28	1712	0.0301	chr6:108214754	10	GT / G	1
*SEC63***	NP_009145.1:p.Lys530GlufsTer30	510	0.0256	chr6:108214773	10	C / CT	2
*SYNJ2***	NP_003889.1:p.Pro1113LeufsTer5	1612	0.0256	chr6:158508008	8	AC / A	1
*GRB14***	NP_004481.2:p.Lys297AsnfsTer23	4231	0.0239	chr2:165365287	9	GT / G	1
*LTN1***	NP_056380.3:p.Asn536MetfsTer33	1016	0.0236	chr21:30339205	11	AT / A	2
*SYNJ2***	NP_003889.1:p.Pro1113LeufsTer5	795	0.0235	chr6:158508008	8	AC / A	2
*PRRT2***	NP_660282.2:p.Arg217ProfsTer8	862	0.0218	chr16:29825015	10	G / GC	1
*LTN1***	NP_056380.3:p.Asn536MetfsTer33	2980	0.0213	chr21:30339205	11	AT / A	1
*TGFBR2***	NP_003233.4:p.Lys128SerfsTer35	1026	0.0212	chr3:30691871	10	GA / G	2
*RGS22***	NP_056483.3:p.Leu1163IlefsTer32	3135	0.0204	chr8:100990177	10	A / AT	1

*Top FSMs with DAF > 0.05 in brain tumor and **Top FSMs with DAF > 0.02 in cfDNA.
Depth: The total high quality unique molecule depth covering the variant. DAF: Deep Allele Frequency. The allele fraction of the reads from deep (i.e. error-correctable) molecular bins that support the alternative allele (DAO/DDP). HRUN: Homopolymer count. The maximum homopolymer length of the variant varies according to the reference (Ref) and alternative (Alt) sequence.

Supplemental Table 4. Patient and specimen description and number of FSMs detected in bone marrow and cfDNA of LS patients

**Characteristics and specimen**	**LS01**	**LS02**	**LS03**
Syndrome	Lynch	Lynch	Lynch
Affected gene	*PMS2*	*PMS2*	*MLH1*
Germline mutation	Deletion exons 2-9	c.137G>T	c.2059C>T
Gender	Male	Male	Female
Race	White	White	White
Blood specimen	n.a.	Plasma	Plasma
Collection Date in reference to diagnosis date	Diagnostic specimen	Diagnostic specimen	Diagnostic specimen
Age at Dx of malignancy	17 mo	15 y	2.5 y
Age at Sample Collection	17 mo	15 y	2.5 y
Approx. amount of plasma available	n.a.	1 mL	1 mL
Diagnosis of malignancy	MDS BM with excess blasts; RAEB	T-ALL with *BCL11B::TLX3* fusion	B-ALL with *ETV6::RUNX1* fusion
Blasts %	4%	92%	75%
Tissue specimen	Bone marrow (BM)	Bone marrow (BM)	Bone marrow (BM)
Therapy prior to sample collection	None	None	None
# FSMs in BM	9	23	33
# Genes in BM (% of genes in the panel)	9 (7%)	23 (19%)	26 (21%)
# FSMs in cfDNA	n.a.	8	10
# Genes in cfDNA (% of genes in the panel)	n.a.	8 (7%)	10 (8%)

n.a.: not available; Dx: diagnosis; MDS: myelodysplastic syndrome; BM: bone marrow; RAEB: refractory anemia with excess blasts; T-ALL: T-cell acute lymphoblastic leukemia; B-ALL: B-cell acute lymphoblastic leukemia; mo: months; y: years.
